# Illuminating glioma surgery: A meta-analysis of Raman spectroscopy for intraoperative decision-making

**DOI:** 10.1007/s10143-025-03836-z

**Published:** 2025-10-31

**Authors:** Gianluca Trevisi, Matteo Palermo, Paolo Barbone, Carmelo Lucio Sturiale

**Affiliations:** 1 Department of Neurosciences, Imaging and Clinical Sciences, G. D’Annunzio University, Chieti-Pescara, Italy; 2 Neurosurgical Unit, Ospedale Civile Spirito Santo, Pescara, Italy; 3 Department of Neurosurgery, Fondazione Policlinico Universitario A. Gemelli IRCCS, Università Cattolica del Sacro Cuore, Rome, Italy; 4 Institute of Neurosurgery, Università Cattolica del Sacro Cuore, L.go A. Gemelli 8, Rome, 00168 Italy

**Keywords:** Border, Glioma, Grading, IDH, Intraoperative, Raman spectroscopy, Surgery

## Abstract

Precise tumor characterization is key in glioma surgery. Raman spectroscopy offers real-time, molecular-level tissue analysis for intraoperative guidance. This meta-analysis synthesizes current applications of Raman spectroscopy in glioma surgery. Systematic literature search in PubMed/MEDLINE and the Cochrane Library through March 15, 2025, using the algorithm: *“Raman AND (brain tumor OR glioma OR glioblastoma) AND (surgery OR intraoperative)”* retrieved 206 studies. Studies evaluating intraoperative Raman spectroscopy for glioma diagnosis, using histopathology/molecular biology as the reference standard, were included. Meta-analysis, following PRISMA-DTA/STARD guidelines, compared Raman spectroscopy to histopathology/molecular biology. Pooled analyses assessed Raman spectroscopy’s ability to: (1) delineate tumor margins; (2) grade high-grade (HGG) versus low-grade gliomas (LGG); (3) distinguish astrocytomas from oligodendrogliomas; (4) classify IDH-wildtype (IDHwt) versus IDH-mutant (IDHmut) gliomas; and (5) discriminate gliomas from other brain tumors. From 206 studies, 19 were used in pooled analyses. For tumor vs. normal tissue, pooled sensitivity was 95.66%, and specificity was 86.13%, with heterogeneity due to varying definitions of *“normal”* tissue. Differentiation of HGG vs. LGG had Limited data; one study showed 91% sensitivity and 87% specificity. Astrocytoma vs. oligodendroglioma differentiation showed a pooled sensitivity of 89.9% and specificity of 86.4%. IDHwt vs. IDHmut glioma classification had a pooled sensitivity of 91.4% and specificity of 90.4%. For glioma vs. other brain tumors, pooled sensitivity was 91.2% and specificity was 91.6%. Raman spectroscopy shows potential for intraoperative glioma characterization. Standardization is needed for better comparison across studies.

## Introduction

Optimizing patient outcomes in glioma surgery depends on precise tumor characterization and resection [1]. Beyond accurate tumor border identification, surgical planning, including the degree of surgical aggressiveness, can also be influenced by specific tumor and patient characteristics [2]. Furthermore, preoperative imaging may struggle to differentiate gliomas from other intracranial pathologies, necessitating distinct surgical approaches [3].

Raman spectroscopy, a technique leveraging the inelastic scattering of photons (Raman shift), offers a powerful solution by providing real-time, molecular-level tissue analysis [4]. By illuminating tissues with a focused laser and analyzing the resulting spectral shifts, a ‘virtual histology’ can be generated, directly informing intraoperative decision-making.

This review synthesizes current applications of intraoperative Raman spectroscopy in glioma surgery, focusing on its capacity to: delineate tumor margins, grade HGG versus LGG, distinguish between astrocytomas and oligodendrogliomas, classify IDHwt versus IDHmut gliomas, and discriminate gliomas from other brain tumors.

## Materials and methods

This meta-analysis followed PRISMA-DTA and STARD guidelines [5, 6], following specific suggestions for meta-analyses of diagnostic accuracy studies [7, 8].

A systematic search was conducted in PubMed/MEDLINE, Cochrane Library, and major trial registries through March 15, 2025, using the search algorithm: *Raman AND (brain tumor OR glioma OR glioblastoma) AND (surgery OR intraoperative)*. Reference lists were also screened.

Studies were included if they evaluated intraoperative Raman spectroscopy for glioma diagnosis in humans using histopathology/molecular biology as the reference standard.

Reviews, case reports, non-English articles, studies not pertinent or lacking detailed glioma data, or those using incorrect reference standards were excluded. For pooled analysis, studies needed sufficient data to extract True Positive (TP), False Negative (FN), False Positive (FP), and True Negative (TN) counts.

Two authors independently screened titles, abstracts, and full texts, resolving disagreements by consensus.

Data extracted included study characteristics, patient details, Raman technical specifics, and TP, FN, FP, TN counts for five predefined diagnostic aims: (1) glioma vs. normal; (2) HGG vs. LGG; (3) astrocytoma vs. oligodendroglioma; (4) IDHwt vs. IDHmut; and (5) glioma vs. other tumors.

Study quality and applicability were assessed using the QUADAS-2 tool [9].

Counts were pooled using a bivariate random-effects model to calculate summary Sensitivity, Specificity, Positive and Negative Likelihood Ratios, and Diagnostic Odds Ratio (DOR). Heterogeneity was assessed using the I² index. Analyses used OpenMetaAnalyst software.

## Results

### Literature search

**Fig. 1:**
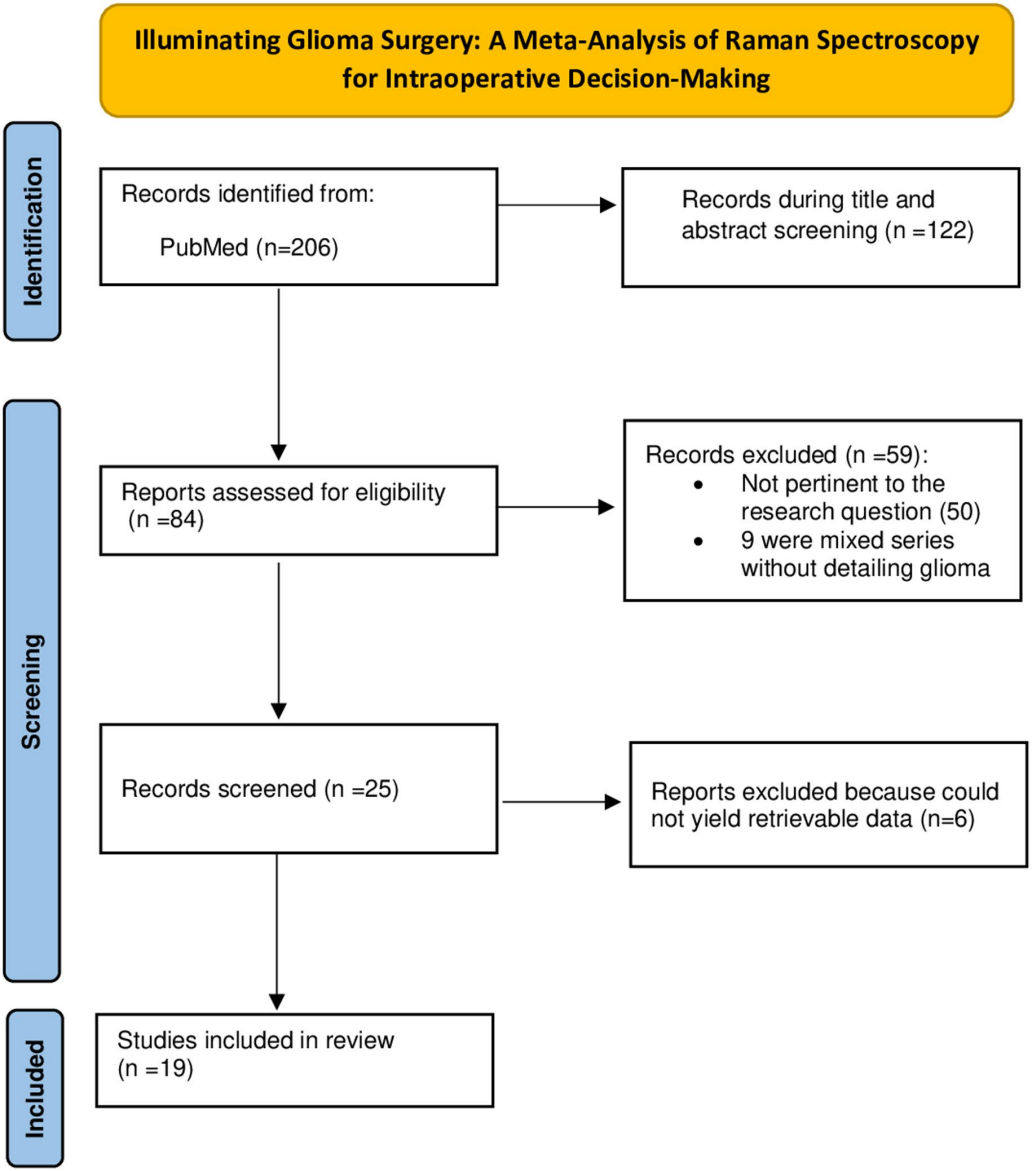
Flow chart of the search for eligible studies on the diagnostic performance of Raman Spectroscopy in glioma surgery

The search strategy identified 206 studies. After reviewing titles and abstracts, 122 studies were excluded: 84 were not pertinent to the research question or did not involve human subjects, 34 were reviews, editorials, or letters, and 4 were not in English. The full texts of the remaining 84 papers were retrieved and assessed for eligibility. Subsequently, 59 articles were excluded: 50 were not pertinent to the research question, and 9 presented mixed tumor series without detailed glioma data. Of the remaining 25 papers, 19 were suitable for the systematic review and pooled analysis for at least one aim. Six studies were pertinent to the qualitative analysis but lacked sufficient data to recalculate true-positive (TP), true-negative (TN), false-positive (FP), and false-negative (FN) values, and therefore could not be included in any pooled analyses. Figure 1 summarizes the literature search process.

## Systematic review and pooled analysis

This systematic review and meta-analysis evaluated the diagnostic accuracy of Raman spectroscopy across several glioma classification tasks. For each aim, retrieved papers are individually reported. Data from papers that explicitly report pooled estimates were calculated for sensitivity, specificity, likelihood ratios, diagnostic odds ratio (DOR), and the area under the curve (AUC) of the summary receiver operating characteristic (sROC) curve. The main results of the meta-analysis are summarized in Table 1. The QUADAS-2 tool was used for quality assessment (Table 2).

**Table 1:** Summary of study characteristics and data for pooled analyses (Refer to text for aim Definitions)

Aim	First Author & Year	Study Characteristics	Technical Aspects	*N* (Condition 1/Condition 2)	Unit for Metrics	TP	FN	FP	TN	Sens (%)	Spec (%)	PPV (%)	NPV (%)	DA (%)
1	Leslie et al. 2012	Mixed Prosp/Retro (Pediatric)	Raman microscopy (785 nm), fresh/frozen tissue, SVM	40 cases (24 G/16 N)	Spectra	127	5	5	159	96.2%	97%	96.2%	97%	96.6%
1	Ji et al. 2015	Prospective	SRS microscopy, fresh tissue, quantitative analysis, GAM	22 pts (15 G/3 N epilepsy controls)	FOVs	140	11	0	74	92.7%	100%	100%	87.1%	95.1%
1	Liu et al. 2016	Prospective	Confocal Raman (785 nm), ex vivo tissue, LVQ neural network	20 pts (20 G)	Spectra	17	2	2	12	89.5%	85.7%	89.5%	85.7%	87.9%
1	Galli et al. 2019	Prospective	Raman microscopy (785 nm), fresh biopsies, PCA-QDA	104 Biopsies (87 G/17 N)	Biopsies	87	0	10	7	100%	41.2%	89.7%	100%	90.4%
1	Sun et al. 2019	Retrospective	SERS (785 nm), tissue supernatant, PLS	23 samples (11 G/12 N)	Spectra	11	0	0	12	100%	100%	100%	100%	100%
1	Klamminger et al. 2021	Training/Validation cohort	Raman spectroscopy (785 nm), FFPE tissue, SVM	99 points (62 G/37 PeriT)	Measuring points	58	4	17	20	93.5%	54.1%	77.3%	83.3%	78.8%
1	Pekmezci et al. 2021	Prospective	Stimulated Raman Histology (SRH), fresh margin specimens	31 pts (31 G) ->128 Specimens	Margin Specimens	66	6	3	53	91.7%	94.6%	95.7%	89.8%	93%
1	Riva et al. 2021	Prospective	Raman spectroscopy (785 nm), fresh biopsies, Gradient Boost	63 samples (38 G/25 N) ->63 Spectra	Spectra	35	3	8	17	92.1%	68%	81.4%	85%	83.9%
1	Jabarkheel et al. 2022	Prospective (Pediatric)	Raman spectroscopy (Solais, 785 nm), fresh ex vivo, PCA + LogReg	415 Spectra (196 G/219 N)	Spectra	179	17	41	178	91.3%	81.3%	81.4%	91.3%	86.1%
1	Herta et al. 2023	Prospective (GBM only)	In vivo Raman (Sentry S1000 probe), peritumoral, classifier	185 Samples (19 G/166 N)	Samples	12	7	18	148	63.2%	89.2%	40%	95.5%	86.5%
1	Zhang et al. 2023	Prospective	Handheld Visible Resonance Raman (VRR), fresh, PCA-SVM	359 Spectra (333 G/26 N)	Spectra	331	2	13	13	99.4%	50%	96.2%	86.7%	95.9%
1	Ember et al. 2024	Prospective, Multicenter	In situ Raman (Sentry System, 785 nm), SVM	518 Spectra (175 G/343 N)	Spectra	159	16	31	312	90.9%	90.9%	83.7%	95.1%	90.9%
1	Li & Chen 2024	Prospective	Miniature Raman (532 nm), fresh, Digital Multimodal + CNN	411 Spectra (260 G/151 N)	Spectra	251	9	29	122	96.5%	80.8%	89.6%	93.1%	90.7%
1	He et al. 2025	Prospective	Raman (532 nm), fresh tissue, CNN	490 Spectra (267 G/223 N)	Spectra	257	10	19	204	96.2%	91.5%	93.1%	95.3%	94.1%
1	Leblond et al. 2025	Retrospective + New cases	In situ Raman (Sentry System, 785 nm), SVM generalizability	248 Spectra (100 G/148 N)	Spectra	100	0	28	120	100%	81.1%	78.1%	100%	88.9%
2	Leslie et al. 2012	Mixed Prosp/Retro (Pediatric)	Raman microscopy (785 nm), fresh/frozen tissue, SVM	246 Spectra (128 HGG/118 LGG)	Spectra	116	12	15	103	90.6%	87.3%	88.5%	89.6%	89%
3	Leslie et al. 2012	Mixed Prosp/Retro (Pediatric)	Raman microscopy (785 nm), fresh/frozen tissue, SVM	75 Spectra (55 Astro/20 Oligo)	Spectra	51	4	0	20	92.7%	100%	100%	83.3%	94.7%
3	Galli et al. 2019	Prospective	Raman microscopy (785 nm), fresh biopsies, PCA-QDA	21 Pts (11 Astro IDHmut/10 Oligo)	Biopsies	8	3	1	9	72.7%	90%	88.9%	75%	81%
3	Leblond et al. 2025	Retrospective + New cases	In situ Raman (Sentry System, 785 nm), SVM generalizability	100 Spectra (31 Astro/69 Oligo)	Spectra	31	0	13	56	100%	81.2%	70.5%	100%	87%
4	Livermore et al. 2021	Prospective	Raman spectroscopy (785 nm), fresh tissue, PCA-LDA	120 Samples (60 IDHwt/60 IDHmut)	Samples	55	5	3	57	91.7%	95%	94.8%	91.9%	93.3%
4	Nohman et al. 2024	Prospective	SRH + AI classifier (DeepGlioma)	102 Scans (3 IDHmut/99 IDHwt)	Scans	3	0	14	85	100%	85.9%	17.6%	100%	86.3%
5	Galli et al. 2019	Prospective	Raman microscopy (785 nm), fresh biopsies, PCA-QDA	380 Spectra (261 GBM/119 Mets)	Spectra	212	49	24	95	81.2%	79.8%	89.8%	65.9%	80.8%
5	Reinecke et al. 2024	Prospective Multicenter	SRH + Deep Learning (RapidLymphoma)	420 (395 Glioma IDH-w/25 PCNSL)	Patients	359	36	0	25	90.9%	100%	100%	41%	91.4%
5	Scheffler et al. 2025	Retrospective	SRH + AI (CTransPath)	40 Images (21 GBM/19 PCNSL)	Images	21	0	3	16	100%	84.2%	87.5%	100%	92.5%

Astro (Astrocytoma), AUC (Area Under Curve), CNN (Convolutional Neural Network), CNSL (Central Nervous System Lymphoma), DA (Diagnostic Accuracy), FFPE (Formalin-Fixed, Paraffin-Embedded), FOVs (Fields of View), G (Glioma), GAM (Generalized Additive Model), GBDT (Gradient Boosting Decision Trees), GBM (Glioblastoma), GTR (Gross Total Resection), HGG (High-Grade Glioma), IDHmut (IDH-Mutant), IDHwt (IDH-Wildtype), LGG (Low-Grade Glioma), LVQ (Learning Vector Quantization), Mets (Metastases), N (Normal), N/A (Not Applicable), NPV (Negative Predictive Value), N/S (Not Specified), NW (Normal White Matter), Oligo (Oligodendroglioma), PCA (Principal Component Analysis), PCNSL (Primary CNS Lymphoma), PeriT (Peritumoral), PLS (Partial Least Squares), PPV (Positive Predictive Value), Prosp/Retro (Prospective/Retrospective), Pts (Patients), QDA (Quadratic Discriminant Analysis), SERS (Surface-Enhanced Raman Scattering), Sens (Sensitivity), Spec (Specificity), SFSA (Spatial Frequency Spectroscopy Analysis), SRH (Stimulated Raman Histology), SRS (Stimulated Raman Scattering), SVM (Support Vector Machine), VRR (Visible Resonance Raman)

**Table 2:** Risk of bias

Paper	Risk of Bias				Applicability concerns		
	Patient Selection	Index Test	Reference Standard	Flow and Timing	Patient Selection	Index Test	Reference Standard
Leslie et al. (2012)	Unclear	Low	Low	Unclear	Low	Low	Low
Ji et al. (2015)	Low	Low	Low	Low	Low	Low	Low
Liu et al. (2016)	Unclear	Unclear	Unclear	Unclear	Unclear	Unclear	Unclear
Sun et al. (2019)	Unclear	Unclear	Unclear	Unclear	Unclear	Unclear	Unclear
Galli et al. (2019)	Low	Low	Low	Low	Low	Low	Low
Pekmezci et al. (2021)	Low	Low	Low	Low	Low	Low	Low
Riva et al. (2021)	Low	Low	Low	Low	Low	Low	Low
Klamminger (2021)	Low	Low	Low	Low	Low	Low	Low
Livermore et al. (2021)	Low	Low	Low	Low	Low	Low	Low
Jabarkheel et al. (2022)	Low	Low	Low	Low	Low	Low	Low
Herta et al. (2023)	Low	Low	Low	Low	Low	Low	Low
Zhang et al. (2023)	Unclear	Low	Low	Unclear	Low	Low	Low
Ember et al. (2024)	Low	Low	Low	Low	Low	Low	Low
Li & Chen (2024)	Unclear	Unclear	Unclear	Unclear	Unclear	Unclear	Unclear
Nohman et al. (2024)	Unclear	Low	Unclear	Unclear	Unclear	Low	Unclear
Reinecke et al. (2024)	Low	Unclear	Unclear	Unclear	Low	Unclear	Unclear
He et al. (2025)	Unclear	Unclear	Unclear	Unclear	Unclear	Unclear	Unclear
Leblond et al. (2025)	Low	Low	Low	Low	Low	Low	Low
Scheffler et al. (2025)	Low	Unclear	Unclear	Unclear	Low	Unclear	Unclear

### Aim 1: tumor vs. normal tissue differentiation

Precisely differentiating tumor from normal brain tissue intraoperatively remains a critical challenge for maximizing tumor resection while preserving neurological function. Raman spectroscopy has emerged as a promising tool to address this, leveraging subtle biochemical differences detectable through spectral analysis. Numerous studies have investigated various Raman-based techniques – including spontaneous Raman, Stimulated Raman Scattering (SRS), Surface-Enhanced Raman Scattering (SERS), Stimulated Raman Histology (SRH), Diffuse Reflectance Spectroscopy (DRS), and Visible Resonance Raman (VRR) – coupled with diverse analytical methods like PCA-LDA, neural networks (LVQ, CNN), PLS, SVM, and Gradient Boosting Decision Trees (GBDT) to distinguish glioma from non-neoplastic tissue.

Research has spanned over a decade, exploring applications from pediatric tumors to adult gliomas, utilizing fresh, frozen, and even formalin-fixed paraffin-embedded (FFPE) samples, and employing both benchtop systems and handheld probes. While many studies reported high classification accuracies, a significant limitation across this body of work is the considerable heterogeneity in the definition and source of the “normal” or control brain tissue used for comparison. Control samples ranged from potentially altered tissue resected during epilepsy surgery, to peritumoral tissue variably defined and potentially containing microscopic tumor infiltration or reactive changes, to unspecified “normal” tissue sources [10, 11]. This variability complicates direct comparison of results and represents a key challenge in interpreting the Literature. From the studies assessing glioma versus normal tissue differentiation suitable for quantitative synthesis, data from 15 were ultimately included in the overall pooled analysis presented below.

### Studies utilizing tissue from non-tumor related surgeries

Several studies used brain tissue from non-tumor surgeries, often epilepsy resections, as the “normal” comparator. Leslie et al. (2012) assessed Raman spectroscopy for pediatric brain tumors, achieving 92% overall accuracy differentiating tumor from normal brain tissue (from epilepsy surgery) using PCA-LDA analysis on fresh/frozen samples [12]. In 2015, Ji et al. employed stimulated Raman scattering (SRS) microscopy on human glioma specimens compared against epilepsy surgery controls, demonstrating near-perfect histological agreement by visualizing tumor infiltration based on chemical contrast related to cellularity, axonal density, and protein/lipid ratio [13]. Galli et al. (2019) used near-infrared Raman and fluorescence spectroscopy on biopsies from 209 patients, effectively discriminating various brain tumors from healthy brain tissue (epilepsy controls) based on distinct spectral signatures [14].

### Studies utilizing peritumoral tissue as control

A large group of studies defined “normal” tissue as samples taken from the peritumoral region during glioma resection, based on criteria like surgical inspection, imaging, distance, or histology. Liu et al. (2016) analyzed Raman spectra (785 nm) from ex vivo glioma and normal white matter (peritumoral) using a Learning Vector Quantization (LVQ) neural network, reaching diagnostic accuracies of 89.5% for glioma and 85.7% for normal tissue [15]. Klamminger et al. (2021) applied Raman spectroscopy to FFPE glioblastoma samples, using SVM classifiers to differentiate the tumor core, necrosis, and histologically defined peritumoral zones [16]. Pekmezci et al. (2021) used stimulated Raman histology (SRH) to identify residual tumor at the infiltrative margin in unprocessed tissues [17]. Riva et al. (2021) also employed machine learning models on Raman spectra from fresh glioma tissue samples, including peritumoral areas, demonstrating potential for accurate classification [18]. Herta et al. (2023) compared Raman spectroscopy and 5-ALA fluorescence for detecting glioblastoma infiltration, reporting lower accuracy for Raman (63%) than some studies, especially in peritumoral tissue, though both identified the tumor core well [19]. Zhang et al. (2023) used a handheld visible resonance Raman (VRR) analyzer with PCA-SVM on fresh tissue, achieving 97.0% sensitivity and 50.0% specificity differentiating overall cancer from normal samples (peritumoral) [20]. Ember et al. (2024) reported high in situ detection accuracies (91% for glioblastoma) in a multicenter study using the Sentry System (Raman spectroscopy with machine learning), presumably using peritumoral tissue as the comparator during surgery [21]. Li and Chen (2024) investigated digital multimodal spectra with deep learning on miniature Raman spectrometer data, suggesting potential for portable intraoperative tools using peritumoral comparisons [22]. Most recently, Leblond et al. (2025) assessed the generalizability of a Raman machine learning model (developed from the Ember et al. cohort) on different patient groups and tumor types compared against adjacent tissue [23].

### Studies utilizing unspecified or other control definitions

Some studies used “normal” tissue without clearly specifying its source or employed comparisons less suitable for direct pooling. Sun et al. (2019) applied surface-enhanced Raman scattering (SERS) to supernatants from glioma and unspecified “normal” tissues, achieving high discrimination accuracy (up to 100%) with PLS and BPNN models and highlighting 2HG as a potential biomarker [24]. Bury et al. published studies in 2019 (handheld SERS on smears) and 2020 (Raman on fresh frozen tissue) showing potential for differentiation [25, 26]; however, these could not be included in the specific pooled analysis presented here due to their comparison structures (e.g., specific classes vs. all others, or grouping glioma with meningioma). He et al. (2025) used convolutional neural network-assisted Raman spectroscopy comparing glioma to unspecified “normal” tissue, reporting high accuracy (93.9%) and an AUC of 0.996 [27].

### Synthesis and limitations

This overview highlights the extensive research applying Raman-based techniques for glioma differentiation, but underscores the significant limitation posed by the heterogeneous definitions of “normal” control tissue across studies. This variability introduces challenges for direct comparison and meta-analysis. Acknowledging this, a quantitative synthesis was performed on the subset of studies providing suitable data for comparing glioma versus normal tissue. Data from 15 studies were ultimately included in the overall pooled analysis described next.

### Quantitative synthesis (pooled analysis)

The overall pooled analysis, incorporating data from 15 suitable studies comparing tumor versus normal tissue, yielded a high pooled sensitivity of 95.66% (95%CI: 92.17%−97.64%; I²=73%) and specificity of 86.13% (95%CI: 76.1%−92.37%; I²=88%), which corresponds to excellent overall accuracy as visually confirmed by the sROC curve.

The specific subgroup analysis restricted to the 9 studies that compared tumor tissue only against peritumoral tissue showed similar strong performance, with a pooled sensitivity of 95.03% (95%CI: 88.94%−97.85%; I²=80%) and specificity of 81.06% (95%CI: 70.48%−88.47%; I²=87%). When comparing this peritumor subgroup (*n* = 9) to the smaller subgroup (*n* = 4) using epilepsy surgery controls, the peritumor group’s sensitivity (95.0%) was slightly higher than the epilepsy control group’s (91.0%), while their pooled specificities were quite similar (81.1% vs. 82.0%, respectively). However, the epilepsy control subgroup exhibited extreme heterogeneity and a much wider confidence interval for specificity compared to the peritumor subgroup, indicating greater variability in that group.

**Fig. 2:**
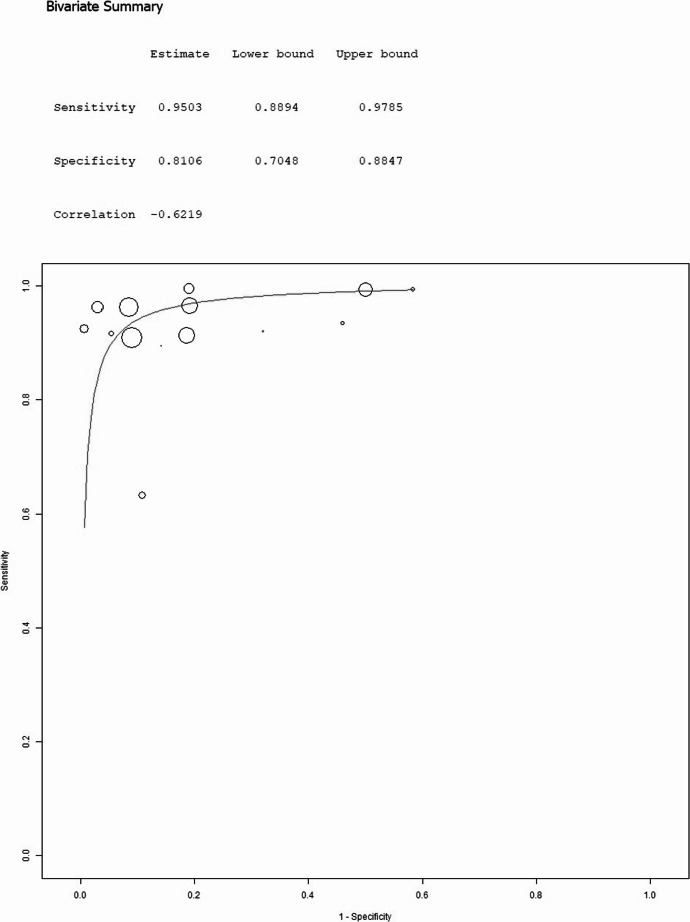
Bivariate summary ROC curve on the diagnostic performance of Raman Spectroscopy in differentiating glioma from normal brain tissue (overall analysis, 15 papers)

**Fig. 3:**
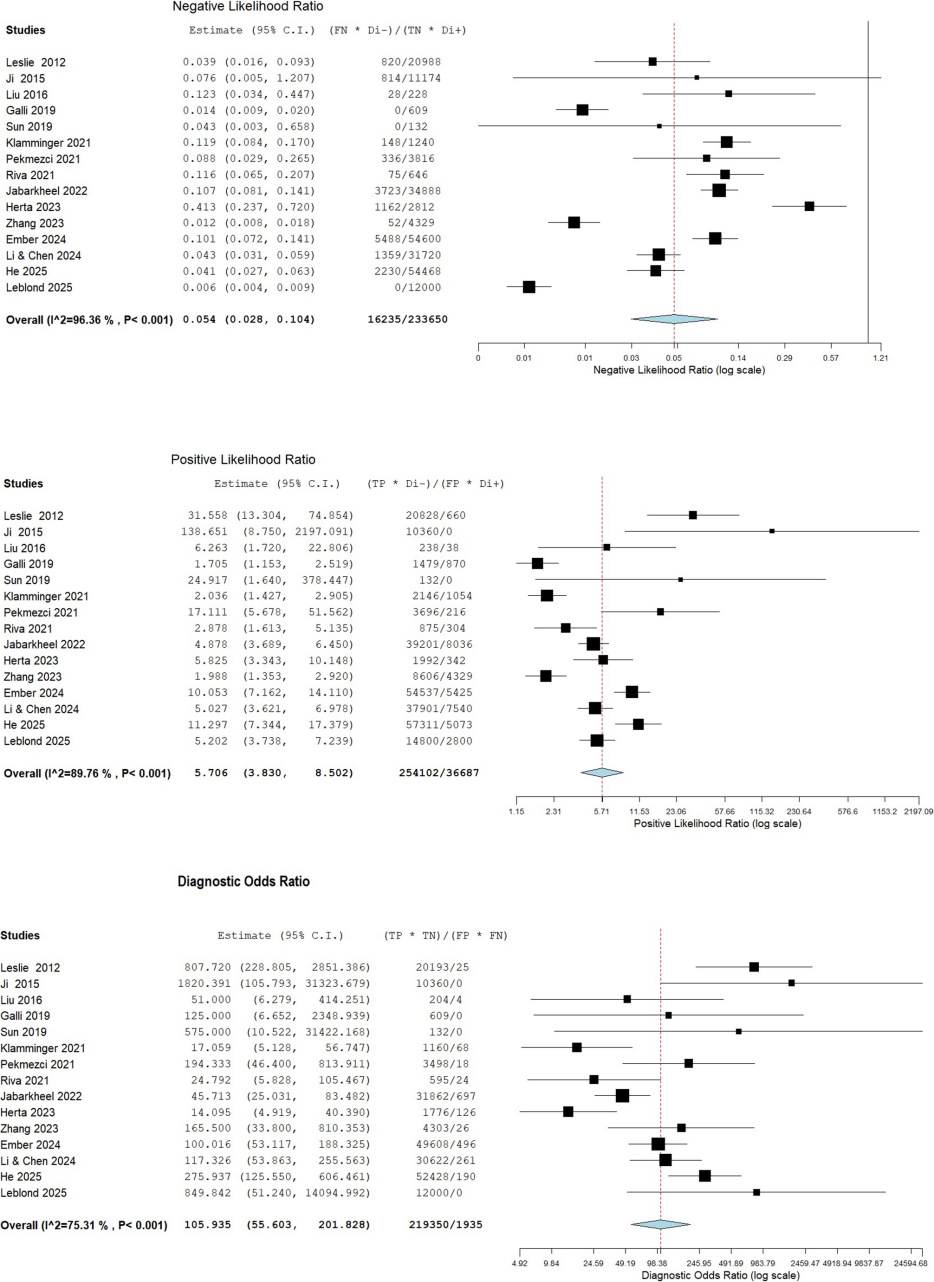
Forest plots of individual studies and pooled negative likelihood ratios, positive likelihood ratios, and diagnostic odds ratios of Raman spectroscopy in differentiating glioma from normal brain tissue (overall analysis, 15 papers), including 95% confidence intervals (95% CI). The size of the squares indicates the weight of each study

**Fig. 4:**
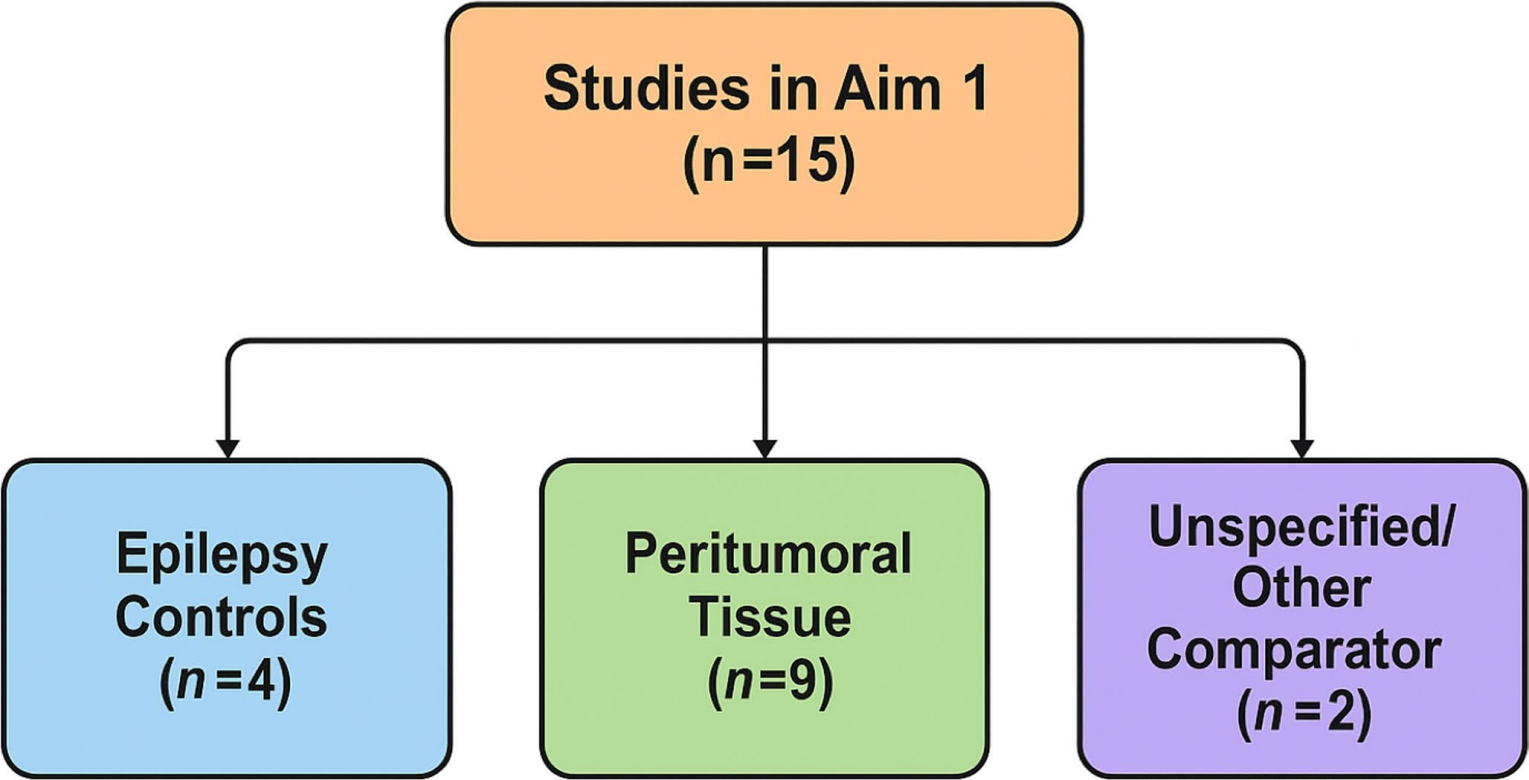
Schematic grouping of included studies for Aim 1 by “normal tissue” comparator

Further diagnostic metrics for the overall analysis (*n* = 15) were: pooled positive likelihood ratio (PLR) of 5.706 (95%CI: 3.830–8.502; I²=90%), pooled negative likelihood ratio (NLR) of 0.054 (95%CI: 0.028–0.104; I²=96%), and pooled diagnostic odds ratio (DOR) of 105.935 (95%CI: 55.603-201.828; I²=75%). For the peritumor only subgroup (*n* = 9), the corresponding metrics were: PLR 4.650 (95%CI: 2.981–7.253; I²=88%), NLR 0.063 (95%CI: 0.025–0.159; I²=97%), and DOR 65.520 (95%CI: 31.658-135.599; I²=68%). Substantial statistical heterogeneity, indicated by high I² values, was observed across studies for several metrics in both the overall analysis and the peritumor subgroup, representing a notable limitation. Tables 1 and 3; Figs. 2 and 3, and 4 summarize the main findings of this aim.

**Table 3:** Pooled analysis of aim 1: overall analysis of 15 papers and subanalysis of 9 papers comparing tumor vs peritumor in the same patients

Tumor Vs Normal Tissue (*n* of papers)	Pooled Sensitivity (95% CI; I^2^)	Pooled Specificity (95% CI; I^2^)	Pooled Negative Likelihood Ratio (95% CI; *p*-value; I^2^)	Pooled Positive Likelihood Ratio (95% CI; *p*-value; I^2^)	Odds Ratio Summary (95% CI; *p*-value; I^2^)
Overall analysis (*n* = 15)	95.66% (92.17%−97.64%; I^2^ **=** 73%)	86.13% (76.1%−92.37%; I^2^ **=** 88%)	0.054 (0.028–0.104; p <.001*; I^2^ **=** 96%)	5.706 (3.830–8.502; p <.001*; I^2^ **=** 90%)	105.935 (55.603-201.828; p <.001*; I^2^ **=** 75%)
Peritumor only (*n* = 9)	95.03% (88.94%−97.85%; I^2^ = 80%)	81.06% (70.48%−88.47%; I^2^ = 87%)	0.063 (0.025–0.159; p <.001*; I^2^ **=** 97%)	4.650 (2.981–7.253; p <.001*; I^2^ **=** 88%)	65.520 (31.658–35.599; p <.001*; I^2^ **=** 68%)

#### Aim 2: high-grade glioma (HGG) vs. low-grade glioma (LGG)

Raman spectroscopy holds promise as a tool for the label-free analysis and classification of brain tumors, including potential differentiation between high-grade (HGG) and low-grade gliomas (LGG). However, studies specifically designed to quantify Raman spectroscopy’s accuracy in distinguishing HGG from LGG are limited.

One such study, by Leslie et al. (2012), evaluated Raman spectroscopy for diagnosing pediatric brain tumors. They reported 91% sensitivity and 87% specificity at the spectral level when differentiating between low-grade gliomas (LGG) and high-grade gliomas (HGG) overall. The study further demonstrated high accuracy for specific glioma subtypes, achieving 100% sensitivity and 96% specificity for classifying low- versus high-grade ependymomas, and 100% sensitivity and specificity for low- versus high-grade oligodendrogliomas. Notably, at the tissue level (classifying the entire sample based on the majority of spectra), perfect separation (100% sensitivity and specificity) was achieved for all these grading comparisons [12]. This was the only study where detailed data on diagnostic accuracy could be retrieved (Table 1). Therefore, we could not perform a pooled analysis for Aim 2. Given that these findings are based on a single study, they should be interpreted as exploratory and require further validation in larger cohorts.

In the study by Li et al. (2025), the authors developed an algorithm to improve the accuracy of brain tumor grading using Raman spectroscopy [28]. A primary challenge they addressed was the difficulty in distinguishing between LGG and HGG due to subtle spectral differences and the low signal-to-noise ratio of Raman spectroscopy. Their algorithm, called ASG-RSFE, incorporates a feature enhancement method using Raman characteristic peak ratios and an adaptive stacking generalization strategy. The authors reported that their model achieved an accuracy exceeding 80% for classifying normal brain tissue, LGG, and HGG. However, this study’s design and data reporting do not allow for its inclusion in the pooled analysis.

#### Aim 3: astrocytoma vs. oligodendroglioma classification

Several studies have explored the use of Raman spectroscopy for differentiating astrocytoma from oligodendroglioma, although data suitable for quantitative synthesis remains limited.

Leslie et al. (2012) investigated pediatric brain tumors using Raman spectroscopy, including astrocytomas and oligodendrogliomas [12]. Although their primary analysis involved multiple glioma subtypes, the data presented allowed for a specific comparison between astrocytoma and oligodendroglioma spectra. Based on the extracted counts, this comparison yielded high performance with a sensitivity of 92.7% and a specificity of 100.0% for distinguishing astrocytoma from oligodendroglioma at the spectral level.

Galli et al. (2019) employed both fluorescence and Raman spectroscopy on fresh biopsies. They achieved approximately 80% accuracy in classifying astrocytoma versus oligodendroglioma with each modality independently. Combining the techniques did not yield further improvement, and the authors noted that misclassified samples often contained features of both tumor types or significant non-neoplastic tissue contamination [14].

Leblond et al. (2025) assessed the generalizability of a pre-trained Raman machine learning model to various tumor types. When applied to astrocytoma and oligodendroglioma, the model showed positive predictive values (PPV) of 70% and 74%, respectively. The authors suggested that incorporating a wider range of Raman biomarkers could enhance detection accuracy [23].

It’s noteworthy that Liu et al. (2024) investigated molecular markers, including 1p/19q status (a key differentiator between IDH-mutant astrocytoma and oligodendroglioma), in high-grade gliomas using Raman spectroscopy [29]. They reported high performance (AUC 0.932) for classifying 1p/19q status itself. However, their study focused on predicting individual molecular markers across various high-grade gliomas and did not provide direct classification data (including the necessary TP/FN/FP/TN counts) specifically for astrocytoma versus oligodendroglioma suitable for inclusion in this pooled analysis.

Based on the data available from the studies by Leslie, Galli, and Leblond that allowed for extraction or calculation of contingency data, a pooled analysis was conducted. The pooled sensitivity for differentiating astrocytoma (defined as the positive class) from oligodendroglioma was 89.9% (95%CI: 68.5%−97.3%), with moderate heterogeneity observed (I²=62%). The corresponding pooled specificity 86.4% (95%CI: 69.3%−94.7%), exhibiting low heterogeneity (I²=29%). Pooled likelihood ratios and the Diagnostic Odds Ratio could not be robustly calculated due to insufficient data reporting within the included source studies.

#### Aim 4: IDH-Wildtype (IDHwt) vs. IDH-Mutant (IDHmut) glioma classification

Raman spectroscopy, coupled with advanced analytical techniques, has emerged as a promising tool for the rapid and accurate classification of gliomas based on their isocitrate dehydrogenase (IDH) mutation status, a critical factor for diagnosis and treatment planning.

Initial investigations by Livermore et al. (2020) [30] demonstrated the feasibility of Raman spectroscopy in distinguishing glioma tissue from normal brain tissue. While not directly focused on IDH status, this study established the foundational potential of Raman spectroscopy for identifying tumor regions suitable for subsequent molecular analysis.

Building upon this, Nohman et al. (2024) [31] explored stimulated Raman histology (SRH) combined with an AI-based classifier for intraoperative IDH mutation prediction. Their model achieved a high level of accuracy, with an area under the receiver operating characteristic curve (AUC) of 0.93 (95%CI: 0.88–0.98; *p* <.0001). This suggests that SRH, integrated with AI, can provide rapid intraoperative IDH status information.

Further advancing this area, Liu et al. (2024) employed deep learning-assisted Raman spectroscopy, specifically a ResNet model, to classify high-grade gliomas based on IDH mutation status. This approach achieved an average AUC of 0.969, with a mean sensitivity of 93.60% and a mean specificity of 92.68%. While these results highlight the potential of deep learning-enhanced Raman spectroscopy for accurate IDH status determination, the absence of a detailed confusion matrix (true positives, false positives, true negatives, false negatives) prevented the calculation of additional diagnostic metrics such as likelihood ratios and DOR.

A pooled analysis of the studies by Livermore et al. and Nohman et al. revealed a pooled sensitivity of 91.4% (95%CI: 81.5–96.2%) and a pooled specificity of 90.4% (95%CI: 75.4–96.7%) for differentiating IDHwt from IDHmut gliomas. The pooled AUC was 0.91. Heterogeneity was low for sensitivity (I²=0%) but moderate for specificity (I²=68%). However, the calculation of pooled likelihood ratios and diagnostic odds ratio (DOR) was precluded by insufficient data reporting within the original studies.

#### Aim 5: gliomas vs. other brain tumors

Raman spectroscopy offers a promising approach for differentiating glioma from other central nervous system (CNS) lesions, particularly metastases and primary CNS lymphoma (PCNSL), a distinction critical for treatment planning.

Acknowledging the inconsistent definition of ‘glioma’ across studies, it is important to note that Reinecke et al. (2024) and Scheffler et al. (2025) focused primarily on high-grade gliomas (IDH-wildtype diffuse glioma or glioblastoma specifically), which may introduce bias when compared with Galli et al. (2019), which employed a broader glioma classification [14, 32, 33]. Furthermore, for the scope of this review, in our analysis glioma was consistently designated as the positive classifier, a methodological choice that may differ from individual study designs.

Regarding differentiation from metastases, Galli et al. (2019) reported using combined fluorescence and Raman data to distinguish glioma from metastases a diagnostic accuracy of 80.9% and a negative predictive value (NPV) of 65.9%, indicative of a notable rate of misclassifying metastases as glioma [14].

For differentiating glioma from PCNSL, advanced techniques combining Stimulated Raman Histology (SRH) with artificial intelligence have shown significant capability.

Reinecke et al. (2024) developed the RapidLymphoma system (SRH with deep learning), achieving high overall balanced accuracy (97.8%) for detecting PCNSL versus other CNS lesions, and similarly high balanced accuracy (> 95%) when comparing PCNSL specifically against IDH-wildtype gliomas or metastases [32]. When analyzing IDH-wildtype glioma (positive class) vs. PCNSL (negative class) differentiation at the patient level, the system showed good sensitivity (~ 91%) and excellent specificity (100%), though the Negative Predictive Value was low (~ 41%) due to some gliomas being misclassified as PCNSL based on overlapping SRH features.

Scheffler et al. (2025) utilized AI model (CTransPath architecture) analyzing SRH images to distinguish PCNSL from glioblastoma, achieving a model accuracy of 92.5%, with 100% sensitivity for glioblastoma and 84.2% specificity for glioblastoma [33].

Pooling data of the studies comparing glioma vs. PCNSL [32, 33], we obtained a sensitivity of 91.2% (95%CI: 85.9%−94.7%; I²=4%) and a specificity of 91.6% (95%CI: 54%−99%; I²=58%).

## Discussion

Raman spectroscopy, a molecular technique analyzing the inelastic scattering of photons by molecules, provides a spectral fingerprint characteristic of a tissue’s biochemical composition, offering a ‘virtual histology’ in real-time. This capability addresses paramount challenges in neurosurgical oncology, particularly the precise intraoperative differentiation and classification of gliomas, which directly impacts resection extent, functional preservation, and patient outcomes. This review synthesized evidence on the application of Raman spectroscopy across several key aims in glioma surgery, revealing both significant promise and areas needing further development.

A recent, relevant meta-analysis by Krzemińska et al. also systematically reviewed the diagnostic accuracy of Raman spectroscopy, confirming its high potential for differentiating major tumor types from normal tissue [23]. Our work provides a distinct and complementary contribution by addressing a broader set of five specific clinical aims, including tumor grading, molecular subtyping (e.g., IDH status), and distinguishing glioma from other entities Like PCNSL. Furthermore, our Literature search extends to a more recent cutoff date of March 15, 2025, and our analysis of heterogeneity focuses specifically on the critical variable of control tissue type (peritumoral vs. epilepsy controls), offering a different perspective than the sample-state analysis performed by Krzemińska et al.

Indeed, a primary aim is the accurate delineation of tumor margins from normal brain tissue, crucial for maximizing cytoreduction while safeguarding function. Other intraoperative tools used for this purpose include imaging techniques like intraoperative MRI and intraoperative ultrasound (iUS). iUS is considered accurate, safe, and cost-effective for estimating the extent of resection, with studies suggesting similar accuracy to iMRI but at a lower cost [34]. Complementary to these structural imaging methods are techniques providing molecular-level information. 5-aminolevulinic acid (5-ALA) fluorescence highlights tumor tissue based on metabolic activity, and Raman spectroscopy offers detailed biochemical analysis. Studies exploring 5-ALA in conjunction with Raman spectroscopy have shown potential for improved accuracy (Leslie et al., 2012 [12]; Galli et al., 2019 [14]). Direct comparisons suggest RS may offer advantages in detecting infiltration, particularly in non-fluorescing or ambiguously fluorescing areas (Herta et al., 2023 [19]). Our pooled analysis demonstrated high diagnostic potential for RS alone, achieving an overall sensitivity of 95.7% and specificity of 86.1% in distinguishing tumor from normal tissue. Similar performance was noted against peritumoral tissue (sensitivity 95.0%, specificity 81.1%). Various RS modalities underpin these results, including spontaneous Raman, Stimulated Raman Histology (SRH) for microscopic imaging [13, 17, 35], Surface-Enhanced Raman Scattering (SERS) [24, 36], Visible Resonance Raman (VRR) [20], and Diffuse Reflectance Spectroscopy (DRS) used alongside Raman [37]. These techniques, often coupled with machine learning, have demonstrated success, even achieving detection at cellular resolution [38]. Near-infrared autofluorescence, frequently acquired concurrently, also offers complementary diagnostic information [39]. Among these approaches, SRH integrated with artificial intelligence has shown particularly high diagnostic accuracy and holds significant translational potential due to its real-time intraoperative applicability. However, significant heterogeneity in defining ‘normal’ controls, especially given the complex and biologically distinct nature of the peritumoral microenvironment [10, 11], and substantial statistical heterogeneity (indicated by high I² values) in pooled analyses limit direct comparisons. Notably, despite the complexity of the peritumoral zone, our quantitative synthesis found that studies using peritumoral tissue as control yielded pooled sensitivity and specificity similar to those using other ‘normal’ tissue definitions, reinforcing the technique’s potential while still highlighting the need for standardization and further generalizability assessment [23].

Distinguishing between high-grade (HGG) and low-grade gliomas (LGG) intraoperatively may influence treatment planning. The literature quantifying RS accuracy for this specific task remains limited. Studies like Leslie et al. (2012) in pediatrics and algorithmic approaches reported by Li et al. (2025) show promise, but subtle spectral differences and technical challenges like low signal-to-noise ratios persist [12, 28]. The paucity of studies suitable for pooled analysis currently hinders a robust quantitative synthesis for this grading application.

The differentiation between astrocytoma and oligodendroglioma, crucial due to differing prognoses and treatment responses Linked to 1p/19q status, represents another area where RS shows potential. Our limited pooled analysis based on three studies [12, 14, 23] indicated good sensitivity (89.9%) and specificity (86.4%), though data availability constrained a more comprehensive assessment.

It is critical to note that the included studies for this aim used varied diagnostic criteria, reflecting the evolution of brain tumor classification. Leslie et al., in 2012, classified tumors based on histology alone, prior to the widespread adoption of molecular markers [12]. In contrast, Galli et al. (2019) and Leblond et al. (2025). according to modern WHO standards, explicitly used molecular data, comparing IDH1-mutant astrocytomas against oligodendrogliomas which were, by definition, also IDH-mutant and 1p/19q co-deleted [14, 23]. This significant variability in classification methodology across the studies contributes to heterogeneity and limits the direct comparison and generalizability of the pooled results. Individual studies report varying accuracies, suggesting further refinement, potentially incorporating broader biomarker sets, is needed.

Rapid molecular classification, particularly determining IDH mutation status, is critical for modern glioma management. Our pooled analysis, based on studies by Livermore et al. and Nohman et al. [30, 31], demonstrated that combining optical techniques (RS/SRH) with AI yields high diagnostic accuracy for differentiating IDHwt from IDHmut gliomas (Sensitivity 91.4%, Specificity 90.4%, AUC 0.91). Heterogeneity was low for sensitivity (I²=0%) but moderate for specificity (I²=68%), and insufficient data reporting precluded calculation of likelihood ratios and DOR. While other significant studies also report high accuracy using related techniques (e.g., Hollon et al. [40] using the SRH-based DeepGlioma system; Liu et al. [29] using deep-learning assisted fiber-optic RS), differences in specific methodologies and reporting formats likely prevented their inclusion in our specific pooled sensitivity/specificity calculation. Nonetheless, the collective evidence, including these highly relevant studies [29, 40], strongly supports the growing potential of these approaches, although rigorous validation remains essential [40].

Finally, discriminating gliomas from other common brain tumors like metastases or PCNSL is essential for appropriate treatment. Misdiagnosis can potentially influence surgical strategies; however, adjuvant therapies are not initiated solely based on Raman spectroscopy data without histological confirmation. Several studies have demonstrated RS’s ability to differentiate between glioma, metastases, and meningioma with high accuracy [14, 21, 39]. Recent advancements using SRH coupled with AI have specifically targeted the challenging differentiation between PCNSL and glioma, achieving high classification accuracy intraoperatively [32, 33]. As with other applications, limitations include the heterogeneity of tumor types and methodologies across studies, often precluding large-scale quantitative synthesis, especially for less common entities or specific techniques.

Looking forward, the clinical translation of Raman spectroscopy will depend on several key refinements. Future directions should focus on improving spectral acquisition speed, further miniaturization of handheld probes for enhanced ergonomics, and seamless integration with existing neuronavigation platforms to correlate molecular data with anatomical imaging in real-time. Prospective, multicenter validation studies using standardized protocols are essential to confirm the diagnostic accuracy and clinical utility of this promising technology.

### Limitations

This meta-analysis highlights the promising potential of Raman spectroscopy for intraoperative glioma characterization. However, several limitations should be considered. A key limitation is the heterogeneity in the definition of “normal” control tissue across studies, which complicates direct comparisons and meta-analysis. This heterogeneity significantly limits the comparability and generalizability of pooled estimates. As shown in Table 2, several studies had unclear risk of bias or applicability concerns regarding patient selection, index test, and/or reference standard. Furthermore, substantial statistical heterogeneity was observed across studies for several metrics, representing a notable limitation. Finally, several studies lacked sufficient data for inclusion in the pooled analyses, which limited the scope of quantitative synthesis.

Finally, findings related to Aims 2, 3, and 4 should be regarded as exploratory, given the small number of available studies suitable for pooling, and several pertinent studies lacked sufficient data for inclusion in the quantitative synthesis.

## Conclusions

In conclusion, Raman spectroscopy holds significant promise as an intraoperative tool to address key challenges in glioma surgery, including tumor delineation, glioma grading, glioma subtyping, IDH status determination and differentiation from other CNS lesions.

## Data Availability

No datasets were generated or analysed during the current study.

## Abbreviations

TP: True Positive
FN: False Negative
FP: False Positive
TN: True Negative
DOR: counts, diagnostic odds ratio
AUC: area under the curve
sROC: summary receiver operating characteristic curve
SRS: Stimulated Raman Scattering
SERS: Surface-Enhanced Raman Scattering
SRH: Stimulated Raman Histology
DRS: Diffuse Reflectance Spectroscopy
VRR: Visible Resonance Raman
GBDT: Gradient Boosting Decision Trees
FFPE: formalin-fixed paraffin-embedded
LVQ: Learning Vector Quantization
SERS: surface-enhanced Raman scattering
HGG: high-grade
LGG: low-grade gliomas
IDHwt: IDH-Wildtype
IDHmut: IDH-Mutant
iUS: intraoperative ultrasound
CNS: central nervous system
PCNSL: primary CNS lymphoma
